# Classification of Individual Finger Movements from Right Hand Using fNIRS Signals

**DOI:** 10.3390/s21237943

**Published:** 2021-11-28

**Authors:** Haroon Khan, Farzan M. Noori, Anis Yazidi, Md Zia Uddin, M. N. Afzal Khan, Peyman Mirtaheri

**Affiliations:** 1 Department of Mechanical, Electronics and Chemical Engineering, OsloMet-Oslo Metropolitan University, 0167 Oslo, Norway; haroonkh@oslomet.no; 2 Department of Informatics, University of Oslo, 0315 Oslo, Norway; farzanmn@ifi.uio.no; 3 Department of Computer Science, OsloMet-Oslo Metropolitan University, 0167 Oslo, Norway; anisy@oslomet.no; 4 Department of Neurosurgery, Oslo University Hospital, 0450 Oslo, Norway; 5 Department of Computer Science, Norwegian University of Science and Technology, 7491 Trondheim, Norway; 6 Software and Service Innovation, SINTEF Digital, 0373 Oslo, Norway; zia.uddin@sintef.no; 7 School of Mechanical Engineering, Pusan National University, Busan 46241, Korea; nasirafzal@pusan.ac.kr; 8 Department of Biomedical Engineering, Michigan Technological University, Houghton, MI 49931, USA

**Keywords:** functional near-infrared spectroscopy (fNIRS), finger-tapping, classification, motor cortex, machine learning

## Abstract

Functional near-infrared spectroscopy (fNIRS) is a comparatively new noninvasive, portable, and easy-to-use brain imaging modality. However, complicated dexterous tasks such as individual finger-tapping, particularly using one hand, have been not investigated using fNIRS technology. Twenty-four healthy volunteers participated in the individual finger-tapping experiment. Data were acquired from the motor cortex using sixteen sources and sixteen detectors. In this preliminary study, we applied standard fNIRS data processing pipeline, i.e., optical densities conversation, signal processing, feature extraction, and classification algorithm implementation. Physiological and non-physiological noise is removed using 4th order band-pass Butter-worth and 3rd order Savitzky–Golay filters. Eight spatial statistical features were selected: signal-mean, peak, minimum, Skewness, Kurtosis, variance, median, and peak-to-peak form data of oxygenated haemoglobin changes. Sophisticated machine learning algorithms were applied, such as support vector machine (SVM), random forests (RF), decision trees (DT), AdaBoost, quadratic discriminant analysis (QDA), Artificial neural networks (ANN), k-nearest neighbors (kNN), and extreme gradient boosting (XGBoost). The average classification accuracies achieved were 0.75±0.04, 0.75±0.05, and 0.77±0.06 using k-nearest neighbors (kNN), Random forest (RF) and XGBoost, respectively. KNN, RF and XGBoost classifiers performed exceptionally well on such a high-class problem. The results need to be further investigated. In the future, a more in-depth analysis of the signal in both temporal and spatial domains will be conducted to investigate the underlying facts. The accuracies achieved are promising results and could open up a new research direction leading to enrichment of control commands generation for fNIRS-based brain-computer interface applications.

## 1. Introduction

Functional near-infrared spectroscopy (fNIRS) is a portable and non-invasive brain imaging modality for continuous measurement of haemodynamics in the cerebral cortex of the human brain [1]. Over the last decade, the method has gained popularity due to its acceptable temporal and spatial resolutions, and its easy-to-use, safe, portable, and affordable monitoring compared to other neuroimaging modalities [2]. fNIRS has been used to monitor a variety of cognitive activities, such as attention, problem-solving, working memory, and gait rehabilitation [3]. The underlying theory behind fNIRS functionality is based on optical spectroscopy and neurovascular coupling [1,4]. Optical spectroscopy uses the interaction of light with matter to measure certain characteristics of molecular structures, while neurovascular coupling defines the relationship between local neuronal activity and subsequent changes in cerebral blood flow due to cerebral activity [5,6,7]. It is known that most of the biological tissue is transparent to the near-infrared range (700–900 nm). The near-infrared window commonly used in fNIRS is 690–860 nm [8]. Haemoglobin is a protein that is responsible for delivering oxygen throughout the body via red blood cells. This protein is the major absorbent within the near-infrared range of light ( def. 700–1100 nm). In summary, the continuous-wave fNIRS machine uses two near-infrared wavelengths to measure the relative change in oxygenated haemoglobin (ΔHbO) and deoxygenated haemoglobin (ΔHbR) in cerebral activation.

The most common brain areas studied in neuroimaging are the cerebral prefrontal and motor cortex, particularly for cognitive and motor tasks [9,10]. Since the beginning of the 19th century, the finger-tapping test has been used in various brain studies to assess the motor abilities and accessory muscular control [11]. Various brain and non-brain signals were obtained during the finger-tapping task to access the motor abilities and differentiated movements. Investigating finger movements is particularly important in the field of the brain-computer interface to decode the neurophysiological signal and generate control commands for external devices [9,12]. Individual finger movements were classified with an average accuracy of 85% using electromyogram (EMG) bio-signals while performing finger-tapping tasks [13]. Similarly, in another study using surface EMG, individual and combined finger movements were classified with an average accuracy of 98% on healthy and 90% in below-elbow amputee persons [14]. These higher classification accuracies of finger movements may be best for prosthetic hand development. Other modalities predicting dexterous individual finger movements include ultrasound imaging from the forehand and differentiating finger movements with a higher precision of 98% accuracy [15]. Most brain imaging modalities are limited to the movement of larger body parts, such as the upper and lower limbs. However, it is essential to decode dexterous functions from brain signals in case where other types of brain imaging are difficult to implement. Among invasive brain signals, electrocorticography (ECoG) was shown to differentiate between individual finger movements with acceptable classification accuracies [12,16,17]. However, to the best of the author’s knowledge, only one study was found during a literature review that utilized noninvasive brain signals, i.e., electroencephalography (EEG) signals, to decode individual finger movements. The study found a broadband power increase and low-frequency-band power decrease in finger flexion and extension data when EEG power spectra were decomposed in principal components using principal component analysis (PCA). The average decoding accuracy over all subjects was 77.11% obtained with the binary classification of each pair of fingers from one hand using movement-related spectral changes and a support vector machine (SVM) classifier.

The prevalent motor execution task in fNIRS-based studies includes tapping of one or more fingers, single hand-tapping, both hand-tapping, right and left finger-tapping and hand-tapping. In the study, left and right index finger-tapping was distinguished with a classification accuracy of 85.4% using features from the vector-based phase and linear discriminant analysis [18]. In [19], three different tasks, i.e., right and left-hand unilateral complex finger-tapping, and foot-tapping, were performed. The classification accuracy achieved using SVM was 70.4% for the three-class problem. In single-trail classification for a motor imaginary with thumb and complex finger-tapping task achieves an average accuracy of 81% by simply changing the combination of a set of channels, time intervals, and features [20]. In [21] thumb and little finger were classified with an accuracy of 87.5% for ΔHbO data. Deep learning approaches are also becoming popular for the classification of these complex finger movements. In a study [22], using conditional generative adversarial networks (CGAN) in combination with convolutional neural networks (CNN), the left finger, right finger, and foot-tapping tasks were differentiated with higher classification accuracy of 96.67%. In one of the recent studies, left and right index finger-tapping were distinguished with a different tapping frequency using multilabeling and deep learning [23]. Different labels were assigned to right and left finger-tapping with different tapping frequencies labels such as rest, 80 bpm, and 120 bpm. With this complex combination using deep learning approach the average classification accuracy achieved was 81%. The aforementioned studies are difficult to compare since different models and finger-tapping exercises were conducted. However, according to the literature, the differentiation of finger movement patterns is very challenging using fNIRS. This fact is supported by legacy studies that show that there is no significant statistical difference between fNIRS signals recorded from primary- and pre-motor cortices during sequential finger-tapping and whole-hand grasping [24]. Furthermore, the dynamic relationship between the simultaneously activated brain regions during the motor task is becoming better understood. An interesting study conducted by Anwar et al. [25,26] describes the effective connectivity of the information flow in the sensorimotor cortex, premotor cortex, and contralateral dorsolateral prefrontal cortex during different finger movement tasks using multiple modalities such as fNIRS, fMRI, and EEG. It was found that there is an adequate bi-directional information flow between the cortices mentioned above. The study also concluded that, compared to fMRI, fNIRS is an attractive and easy to use alternative with an excellent spatial resolution for studying connectivity. In this perspective, multi-modal fNIRS-EEG is also an appealing alternative to fMRI. Hence, it is essential to study the flow and connectivity of individual finger movement from the motor cortex using fNIRS or multi-model integration of EEG-fNIRS. The multi-model EEG-fNIRS integration was shown to enhance classification accuracy [27], increase the number of control commands, and reduce the signal-processing time [4,28].

It has been unclear whether fNIRS signals have enough information to differentiate between individual finger movements. Some underlying limitations of fNIRS may be the reason for this drawback, such as comparatively low temporal resolution (1–10 Hz for commercially available portable devices), depth sensitivity of about 1.5 cm (depending upon source-detector distance, which is typically 3 cm), and spatial resolution up to 1 cm [29]. To shed light on this research area, the study is conducted to investigate the detection of individual finger-tapping tasks using fNIRS. Also, the study is a step forward towards understanding the dynamic relationship between the brain regions that are simultaneously activated during motor tasks. We believe that the advances made in sophisticated machine learning algorithms could help to identify individual finger movements from potential fNIRS signals. This study is structured and reported in accordance with the guidelines published in [30]. The following sections will address materials and methods (Section 2), results and discussion (Section 3) and conclusion (Section 4).

## 2. Materials and Methods

The section on materials and methods describes procedure followed during experimental design, data collection, and processing.

### 2.1. Participants

Twenty-four healthy right-handed participants, 18 males (M) and 6 females (F), selected from random university population participated in the experiment. The ages of the participants were for male (mean age ± standard deviation; range) (M = 30.44±3.03; range: 24–34 years), and female (F = 29.17±3.06; range: 24–34 years). The healthy young participants were selected in the age range of 25–35 years because the frequency of finger-tapping can vary between different age groups. The inclusion criterion for right-handedness was that the participants had to write with the right hand. The participants had normal vision or corrected to normal vision. Exclusion criteria include neurological disorders or limitation of motor abilities in any hands or finger. For ethical statements, please see Section 4.

### 2.2. Instrumentation

A continuous-wave optical tomography machine NIRScout (NIRx Medizintechnik GmbH, Germany) was used to spontaneously acquire brain data at one of the laboratories under the ADEPT (Advanced intelligent health and brain-inspired technologies) research group at Oslo Metropolitan University, Norway. The data acquisition used two wavelengths, i.e., 760 nm $\;(\lambda_{1})$ and 850 nm $\;(\lambda_{1})$ with a sampling rate of 3.9063 Hz.

### 2.3. Experimental Setup and Instructions

**Figure 1:**
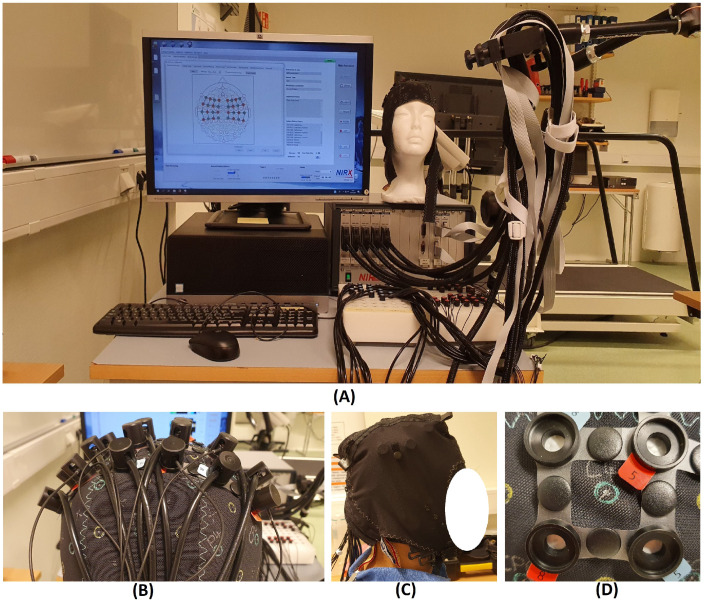
(**A**) Experimental setup; (**B**) optodes arrangement; (**C**) overcap to reduce external light; (**D**) optodes holder.

The experiment was performed in a relatively controlled environment. The environmental light, including monitor screen brightness, was shielded to minimise any influences during stimuli changes in the presentation. A black over-cap was used to further reduce the effect of surrounding light further, as shown in Figure 1C. The experiment was conducted in a noise-free room. A visual presentation of resting and task (finger-tapping corresponding to each finger) was displayed on the computer monitor to the participants. Before starting the actual experiment, the participants were given implicit instructions about the experimental protocol and procedure. Practice sessions were conducted before the experiment. The finger-tapping task was performed at a medium-to-fast pace but not with any specific frequency. The number of repetitions of experiments for each participant was dependent upon the comfort and convenience of the participants. No investigation was conducted during any inconvenience and discomfort experienced by the participant, resulting in unwanted signals such as frustration interference in brain signals. Data were acquired using commercial NIRx software NIRStar 15.1. The complete experimental setup is shown in Figure 1.

### 2.4. Experimental Design

**Figure 2:**
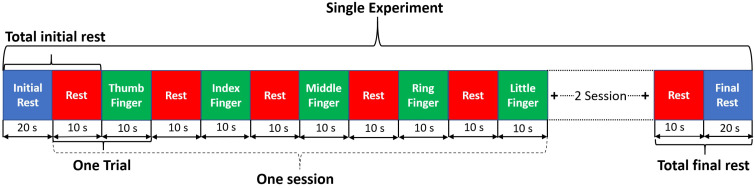
Experimental paradigm visualization. Single experiment consists of three sessions of each finger tapping trail. Single trial consists of 10 s task and 10 s finger tapping.

The experiments were designed using blocks of rest (initial rest, final rest, and inter-stimulus rest) and task (thumb, index middle, ring, and little finger-tapping) of the right hand as shown in Figure 2. An optimal baseline period of 30 s was set up before and after the first and last task, respectively. The stimuli duration necessary to acquire an adequate and robust haemodynamic response corresponding to finger-tapping activity was set to 10 s [31]. The single experimental paradigm consists of three sessions of each finger tapping trial. The total length of an experiment was 350 s. The single trial includes 10 s rest followed by 10 s of the task. Experiments were repeated for each participant from one to three times in a single day depending upon his/her comfortability. The rest and task blocks were presented using NIRX stimulation software NIRStim 4.0.

### 2.5. Brain Area and Montage Selection

**Figure 3:**
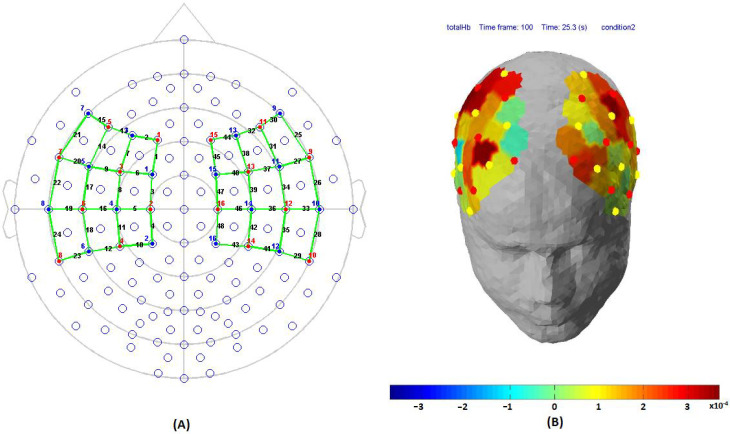
(**A**) Source-detector placement over motor cortex. Figure 3A Colour code: Red (source), Blue (detector), Green (channels), and black colour represent channel numbers. (**B**) Demonstration of total haemoglobin changes over motor cortex during index finger tapping.

Before placing the NIRScap on the participant’s head, cranial landmarks (inion and nasion) were marked to locate $C_{z}$. The emitter and detector were placed in accordance with 10-5 international positioning layout. The distance between source and detector was kept at 3 cm using optode holders. Sixteen emitters and sixteen detectors were placed over the motor cortex in accordance with standard $motor_{16x16}$ montage available in NIRStar v15.2, as shown in Figure 3A,B. The source-detectors were placed over the frontal lobe (F1, F2, F3, F4, F5, F6, F7, and F8), frontal-central sulcus lobe (FC1, FC2, FC3, FC4, FC5, and FC6), central sulcus lobe (C1, C2, C3, C4, C5, C6), central-parietal lobe (CP1, CP2, CP3, CP4, CP5, and CP6), and the temporal-parietal lobe (T7, T8, TP7 and TP8). The data were collected from both the left and right hemispheres for further research work. However, in this particular work, only the channels of the left hemisphere were only further analysed.

### 2.6. Signal Prepossessing

Signal processing was performed using commercial fNIRS data processing software nirsLAB (v2019014) [32] and Matlab^®^. Signal were pre-processed in nirsLAB, for diverse tasks such as removing discontinuities, spikes, and truncation of the data points before and after the first and last stimuli appeared, respectively. Bad channels were identified using the criterion of the gain setting of three and coefficient of variation (CV) of 7.5% in nirsLAB. The coefficient of variation is equal to a hundred times the standard deviation divided by the mean value of the raw data measurements. A large value for CV is an indication of high noise. The gain setting was set to eight for all the data processed. Optical densities were converted into haemoglobin concentration change using Modified Beer–Lambert Law in nirsLAB (see details in Section 2.7).

### 2.7. Modified Beer–Lambert Law (MBLL)

The changes in optical densities were converted using MBLL into ΔHbO (Equation (1a)) and ΔHbR (Equation (1b)). The parameter for MBLL, such as the differential path length factor (DPF) and molar extinction coefficients (using standard W.B Gratzer spectrum) for ΔHbO and ΔHbR, are shown in Table 1. The molar concentration and MVO2Sat value are set as 75 μM and 70%, respectively.

(1a) $$\begin{array}{cc} \Delta HbO^{i}\left(k\right) & =\frac{\left(\varepsilon_{\Delta HbR}^{\lambda_{1}}\frac{\Delta OD^{\lambda_{2}}\left(k\right)}{DPF^{\lambda_{2}}}\right)-\left(\varepsilon_{\Delta HbR}^{\lambda_{2}}\frac{\Delta OD^{\lambda_{1}}\left(k\right)}{DPF^{\lambda_{1}}}\right)}{l^{i}\left(\varepsilon_{\Delta HbR}^{\lambda_{1}}\varepsilon_{\Delta HbO}^{\lambda_{2}}-\varepsilon_{\Delta HbR}^{\lambda_{2}}\varepsilon_{\Delta HbO}^{\lambda_{1}}\right)} \end{array}$$

(1b) $$\begin{array}{cc} \Delta HbR^{i}\left(k\right) & =\frac{\left(\varepsilon_{\Delta HbO}^{\lambda_{2}}\frac{\Delta OD^{\lambda_{1}}\left(k\right)}{DPF^{\lambda_{1}}}\right)-\left(\varepsilon_{\Delta HbO}^{\lambda_{1}}\frac{\Delta OD^{\lambda_{2}}\left(k\right)}{DPF^{\lambda_{2}}}\right)}{l^{i}\left(\varepsilon_{\Delta HbR}^{\lambda_{1}}\varepsilon_{\Delta HbO}^{\lambda_{2}}-\varepsilon_{\Delta HbR}^{\lambda_{2}}\varepsilon_{\Delta HbO}^{\lambda_{1}}\right)} \end{array}$$

where, $\Delta HbO^{i}$ and $\Delta HbR^{i}$: concentration changes of ΔHbO and ΔHbR, ε(λ): extinction coefficient corresponding to wavelengths and haemoglobin concentrations, ΔOD: variation in optical density at $k^{th}$ sample, DPF(λ): differential path length factor, *i*: $i^{th}$ channel pair representation of emitter-detector, $\lambda_{1}$ and $\lambda_{2}$: two working wavelengths of fNIRS system, $\varepsilon_{HbR}^{\lambda_{1}}$, $\varepsilon_{\Delta HbO}^{\lambda_{2}}$, $\varepsilon_{\Delta HbR}^{\lambda_{2}}$ and $\varepsilon_{\Delta HbO}^{\lambda_{1}}$: extinction coefficients of ΔHbO and ΔHbR at two different wavelengths.

### 2.8. Signal Filtration

The spontaneous contamination from physiological and non-physiological noise in fNIRS data, such as heart rate (≃1 Hz), respiration (≃0.2 Hz), Mayer waves (≃0.1 Hz), and very low frequency (≤0.04, VLF) was removed by applying subsequent filters. Non-physiological noise refers to motion artefacts, measurements noise and machine drift due to the temperature changes in the optical system. The stimulation frequency for the given experimental paradigm was (1/20 s = 0.05 Hz). The stable 4th order band-pass Butter-worth filter with a low and high cut-off frequency of 0.01 Hz and 0.15 Hz, respectively [33], was applied to remove the noises. To avoid phase delay in filtering, the built-in MATLAB® command ’filtfilt’ was used. Furthermore, smoothing of the fNIRS signal was done by applying the Savitzky-Golay filter with the optimal order and frame size recommended in [34]. In [34], the recommended filter order and frame size is three and nineteen, respectively, for a frequency band of 0.03–0.1 Hz. We used the same order and frame size because our band of frequencies are quite similar.

### 2.9. Feature Extraction

The most common statistical features (descriptive and morphological) used in fNIRS are signal mean, peak, minimum, Skewness, Kurtosis, variance, median, and peak-to-peak [35,36,37,38]. The window length was set to 10 s, which is equal to the task period. The descriptions of the extracted features are shown in Table 2 from ΔHbO data.

### 2.10. Classification

Eight commonly used classifiers were evaluated to check the robustness of modern machine learning algorithms for decoding dexterous finger movements. The classifiers included Support vector machine (SVM), Random Forest (RF), Decision tree (DT), Adaboost, Quadratic discriminant analysis (QDA), Artificial neural networks (ANN), k-nearest neighbours (kNN), and Extreme Gradient Boosting (XGBoost). The different classifiers’ parameters are shown in Table 3.

### 2.11. Performance Evaluation

Each classifier was mostly evaluated using different performance measures, like accuracy, precision, recall, F1 score, receiver operating characteristic curve/ROC curve, and confusion matrix [39]. All these measures can be derived from the so-called true positives (TP), false positives (FP), true negatives (TN), and false negatives (FN). Reporting single metrics does not give us a complete understanding of the classifier behavior. Hence, it is important to at-least report a few of these parameters to gain a complete understanding of the classifier behaviour. In this study, we have reported accuracy, precision, recall and F1 score. Accuracy is the ratio between correctly classified points to the number of total point. The accuracy gives the probability of correct predictions of the model. However, in the case of highly imbalanced data sets, the model that deterministically classifies all the data as the majority class will yield higher classification accuracy, which makes this measure unreliable. The confusion matrix summarizes the predicted results in table format with visualisation of all the above-mentioned four parameters (TP, FP, TN, FN) of the classifiers. Precision and recall give us an understanding of how useful and complete are the results, respectively. F1 score is the harmonic mean of precision and recall. All these parameters are discussed in the results section, where we discuss the performance of the classifier in decoding individual finger-tapping.

## 3. Results and Discussion

**Figure 4:**
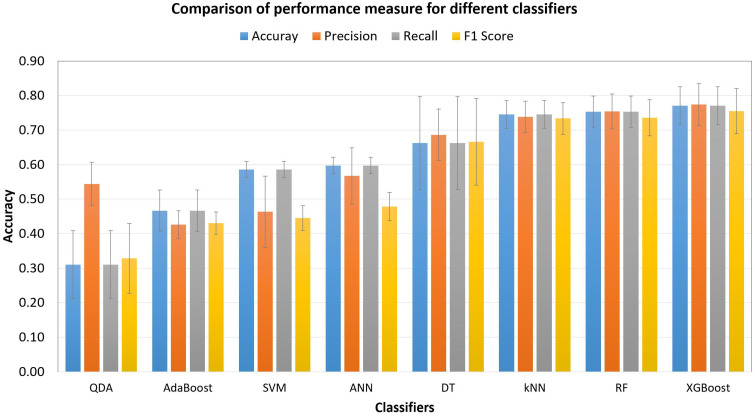
Comparison of different classifiers on basis of performance parameters (accuracy, precision, recall F1score).

**Figure 5:**
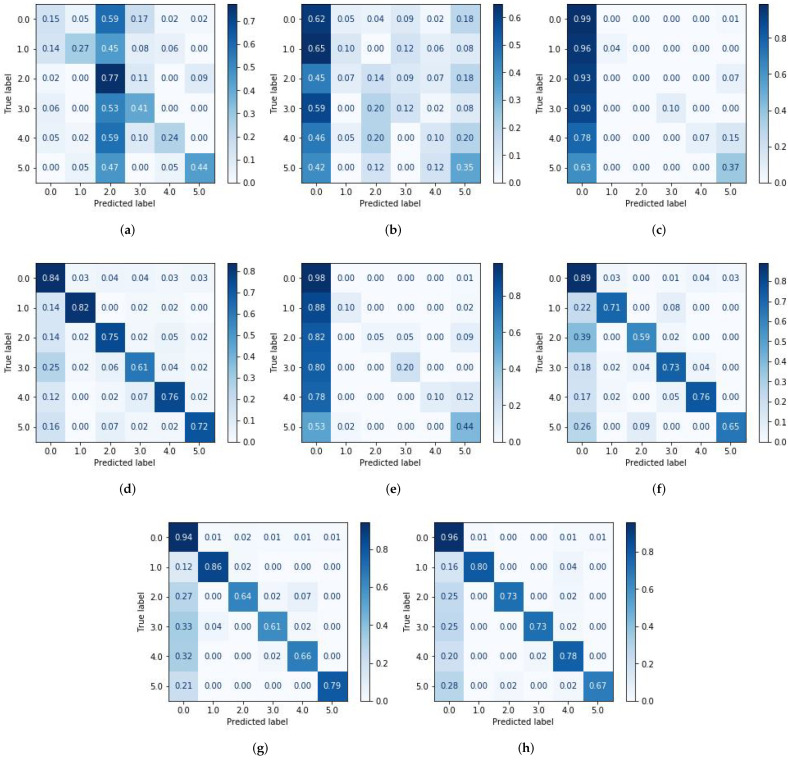
Confusion metrics for all classifiers for subject one (S01); Classes are labeled as ‘0’, ‘1’, ‘2’, ‘3’, ‘4’ and ‘5’, which stands for ‘Rest’, ‘Thumb’, ‘Index’, ‘Middle’, ‘Ring’, and ‘Little’ finger-tapping classes, respectively. (**a**) Quadratic discriminant analysis (QDA). (**b**) AdaBoost. (**c**) Support vector machine (SVM). (**d**) Decision tree (DT). (**e**) Artificial neural networks (ANN). (**f**) k-nearest neighbors (kNN). (**g**) Random forest (RF). (**h**) Extreme Gradient Boosting (XGBoost).

**Figure 6:**
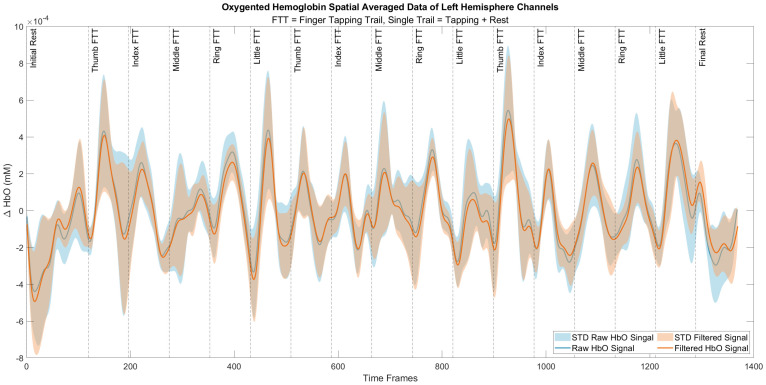
Oxygenated haemoglobin Signal for complete experimental trail.

In this study, we classified individual finger tapping of right-handed people using fNIRS signals. For that purpose, eight different spatio-statistical features were extracted from ΔHbO, as shown in Table 2. Furthermore, we also compared and evaluated the performance of different classifiers, such as SVM, RF, DT, Adaboost, QDA, ANN, kNN and XGBoost, as shown in Figure 4. Table 4 shows the four important performance measures among all of the subjects for the respective classifiers. It was noted that the kNN, RF and XGBoost classifiers yielded maximum classification accuracies, 0.75 ± 0.04, 0.75 ± 0.05, and 0.77 ± 0.06, respectively. We applied the student’s *t*-test to validate whether or not these classifier’s accuracies were statistically discriminant or not with respect to the rest of the classifiers. The *p*-values obtained among kNN, RF, and XGBoost were not statistically significant, since all the classifiers yielded a similar accuracy. On the other hand, the *p*-values using either classifiers kNN, RF or XGBoost versus all of the other classifiers were less than 0.05 for all ΔHbO signals, which establish the statistical significance of these classifiers performance. Previous studies showed that thumb finger-tapping gives a higher level of cortical activation among other fingers [40], which is also supported by our current study as shown in Figure 5f–h. Moreover, the highest peaks in ΔHbO signal which corresponds to higher brain activity during thumb finger-tapping can be seen in Figure 6.

Overall, it was noted that most of the classes were misclassified as a *rest* class, and KNNs were therefore unable to classify the index finger correctly. We tested kNNs on different neighbours (such as 5, 10, and 15), five of which performed better than others, whereas RFs performed poorly on classifying the middle finger. Similarly, like kNNs, we also tested RFs on different estimators and got the best results at 10 number of estimators. On the other hand, XGBoost only classified little fingers poorly. In general, KNNs, RFs, and XGBoost performed well.

One of the core objectives of the brain-computer interface is to achieve a maximum number of commands with good classification accuracy. If we look at the previous literature in the field of fNIRS demonstrates that most of the work utilized either two-class, three-class, or four-class classification. While classifying two commands using fNIRS-based brain signals Power et al. achieved an average classification accuracy of 0.56 for two tasks [41]. Hong et al., achieved an average classification accuracy of 0.75 for three commands [42]. Similarly, several studies have reported classification results for four-class classification as well [43]. To the best of the author’s knowledge, this is the first work that has reported good accuracies for five class-classification in the field of fNIRS. In this work, the achieved classification accuracies are far above the chance level (i.e., 0.2), which shows that machine learning can result in a potential increase in the number of commands in the field of fNIRS-based brain imaging.

In future, the signals will be studied in depth to gain a better understanding and more precise understanding of the cortical hemodynamics response precisely. After all, the attributes of different brain regions and with repetition of trails could vary for the same experimental paradigm [44]. Selection of trails or active channels using the 3-gamma function, changing the window length, detection of initial dip, vector phase analysis, and optimal feature extraction are the future directions for data analysis that could help to increase the classification accuracy. Furthermore, deep learning approaches, including deep belief and convolutional neural networks models, could also help to increase classification accuracy [45]. Moreover, activation of the left and right finger-tapping is dominant in premotor and SMA areas comparative to motor execution finger-tapping [46]. In future work, we will focus on averaging over this region of interest to gain a better idea of which activation regions corresponding to different finger-tapping. Trail-to-trail variability in fNIRS signal for finger-tapping tasks could be reduced using seed correlation methods that can enhance the classification accuracy [47]. We also envisage to using estimation algorithms such as the q-step-ahead prediction scheme and the kernel-based recursive least squares (KRLS) algorithm to reduce the onset delay of the ΔHbO changes due to finger-tapping for real-time implementation in the BCI system [21,48,49,50]. In the study, we considered only ΔHbO data. The reason for selecting ΔHbO is that in the field of fNIRS-based brain imaging, although both ΔHbO and ΔHbR are indicators of cerebral blood flows. However, ΔHbO is more sensitive than ΔHbR [51,52]. As far as ΔHbT and cerebral oxygen exchange COE are concerned, the quantities are dependent on HbO and HbR [53]. In future, ΔHbR and total haemoglobin changes ΔHbT changes will also be considered in ordered to achieve understanding. Moreover, only left hemisphere channels were considered in the study. Investigating the dynamic relationship between the brain regions simultaneously activated during finger-tapping would be an interesting direction for the future study. In recent studies, different stimulation durations were investigated to find the appropriate duration that can shorten the command generation time [54]. Keeping in mind the findings of these studies, shorter stimulation durations will also be investigated in the future.

## 4. Conclusions

Despite the outstanding performance of modern machine-learning algorithms, using functional near-infrared spectroscopy to classify movements from delicate anatomical structures, such as individual finger movements, is very challenging. This work presents a classification of individual finger movements (six classes) from the motor cortex. We have applied eight different classifiers, ranging from simple to sophisticated machine-learning algorithms. Quadratic discriminant analysis (QDA), AdaBoost, Support vector machine (SVM), Artificial neural networks (ANN), and Decision tree (DT) performed poorly, with an average classification accuracy of below 60%. On the other hand, other classifiers such as k-nearest neighbours (kNN), Random forest (RF) and Extreme Gradient Boosting (XGBoost) performed exceptionally well for such high-order data, with an average classification accuracy of 0.75±0.04, 0.75±0.05 and 0.77±0.06, respectively. These are preliminary results from this novel research direction. In future, more in-depth analysis of the temporal and spatial domain will be conducted to understand the signals better. Achieving better classification accuracy could be a quantum leap for control command enrichment in brain-computer interface applications.

## Figures and Tables

**Table 1:** Parameters for Modified Beer–Lambert Law (MBLL).

Wavelength (nm)	DPF (cm)	ΔHbO (1/cm) (moles/L)	ΔHbR(1/cm) (moles/L)
760	7.25	1466.5865	3843.707
850	6.38	2526.391	1798.643

**Table 2:** Spatial feature extracted from ΔHbO.

Sr. No.	Statistical Feature	Mathematical Formulation/Description
1.	Signal Mean	Signal mean is calculated as: $\mu_{w}=\frac{1}{N_{w}}\sum_{k=k_{L}}^{k_{U}}△HbX_{w}$ where, $\mu_{w}$: Mean of window *w*: sample window $N_{w}:$ Number of sample in the window $k_{L}$: Lower limit of the window $k_{U}:$ Upper limit of the window $△HbX_{w}$: Stands for ΔHbO or ΔHbR
2.	Signal Peak (Signal maximum)	The feature select the maximum value in the window.
3.	Signal Minimum	The feature minimum value in the window.
4.	Signal Skewness	Signal skewness is calculated as: $skew_{w}=\frac{E_{x}{\left(\Delta HbX_{w}-\mu_{w}\right)}^{3}}{\sigma^{{}^{3}}}$ where, $E_{x}$ is the expectation, μ is the mean, and σ is the standard deviation of the haemoglobin $\Delta HbX_{w}$
5.	Signal Kurtosis	Signal Kurtosis is calculated as: $Kurt_{w}=\frac{E_{x}{\left(\Delta HbX_{w}-\mu_{w}\right)}^{4}}{\sigma^{{}^{4}}}$ where, $E_{x}$ is the expectation, μ is the mean, and σ is the standard deviation of the haemoglobin $\Delta HbX_{w}$
6.	Signal Variance	Signal variance is the measure of signal spread.
7.	Signal Median	Median is the value separating the higher half from the lower half of values in the time window.
8.	Peak-to-peak	Peak-to-peak is computed as the difference between the maximum to the minimum value in the time window.

**Table 3:** Classifier parameters.

Classifiers	Parameters Setting
QDA	priors = None, reg_param = 0.0
AdaBoost	n_estimator = 10, random_state = 0, learning_rate = 1.0
SVM	Kernal = rbf, degree = 3, random_state = None
ANN	hidden layers = (5, 2), solver=’lbfgs’, random_state = 1, max_liter = 300,
Decision Tree	criterion = entropy, random_state = 0
kNN	n_neighbors = 5
Random Forest	n_estimators = 10, criterion = entropy, random_state = 0
XGBoost	booster = gbtree, verbosity = 1, nthread = maximum number of threads

**Table 4:** Subject-wise comparison of classifiers performance parameters (accuracy, precision, recall, F1 score); ‘S’ stands for subject followed by number.

		S01	S02	S03	S04	S05	S06	S07	S08	S09	S10	S11	S12	S13	S14	S15	S16	S17	S18	S19	S20	S21	S22	S23	S24	Mean	STD
**SVM**	**Accuracy**	0.58	0.57	0.58	0.57	0.56	0.57	0.58	0.59	0.58	0.57	0.57	0.62	0.57	0.57	0.64	0.57	0.59	0.58	0.65	0.60	0.57	0.58	0.60	0.59	0.59	0.02
	**Precision**	0.65	0.32	0.34	0.48	0.32	0.49	0.34	0.62	0.53	0.39	0.41	0.46	0.48	0.41	0.67	0.40	0.35	0.43	0.49	0.44	0.45	0.50	0.52	0.65	0.46	0.10
	**Recall**	0.58	0.57	0.58	0.57	0.56	0.57	0.58	0.59	0.58	0.57	0.57	0.62	0.57	0.57	0.64	0.57	0.59	0.58	0.65	0.60	0.57	0.58	0.60	0.59	0.59	0.02
	**F1 Score**	0.47	0.41	0.43	0.42	0.41	0.42	0.43	0.45	0.45	0.43	0.41	0.49	0.43	0.42	0.55	0.42	0.44	0.43	0.52	0.45	0.42	0.44	0.50	0.46	0.45	0.04
**RF**	**Accuracy**	0.84	0.65	0.84	0.70	0.73	0.77	0.75	0.75	0.75	0.76	0.73	0.73	0.72	0.80	0.78	0.72	0.71	0.75	0.82	0.68	0.77	0.77	0.78	0.78	0.75	0.05
	**Precision**	0.84	0.63	0.85	0.70	0.73	0.77	0.75	0.75	0.75	0.77	0.73	0.73	0.72	0.80	0.80	0.73	0.70	0.75	0.82	0.67	0.77	0.78	0.78	0.78	0.75	0.05
	**Recall**	0.84	0.65	0.84	0.70	0.73	0.77	0.75	0.75	0.75	0.76	0.73	0.73	0.72	0.80	0.78	0.72	0.71	0.75	0.82	0.68	0.77	0.77	0.78	0.78	0.75	0.05
	**F1 Score**	0.83	0.61	0.83	0.67	0.72	0.75	0.73	0.74	0.74	0.75	0.70	0.72	0.70	0.78	0.77	0.70	0.69	0.73	0.81	0.65	0.75	0.76	0.77	0.77	0.74	0.05
**DT**	**Accuracy**	0.79	0.56	0.76	0.28	0.67	0.68	0.23	0.68	0.71	0.70	0.63	0.71	0.65	0.73	0.76	0.71	0.67	0.69	0.76	0.64	0.72	0.71	0.75	0.71	0.66	0.13
	**Precision**	0.79	0.56	0.76	0.49	0.67	0.69	0.53	0.68	0.71	0.70	0.63	0.72	0.65	0.74	0.76	0.71	0.68	0.69	0.78	0.64	0.72	0.72	0.75	0.71	0.69	0.07
	**Recall**	0.79	0.56	0.76	0.28	0.67	0.68	0.23	0.68	0.71	0.70	0.63	0.71	0.65	0.73	0.76	0.71	0.67	0.69	0.76	0.64	0.72	0.71	0.75	0.71	0.66	0.13
	**F1 Score**	0.79	0.56	0.75	0.32	0.67	0.69	0.27	0.68	0.71	0.70	0.63	0.71	0.65	0.74	0.76	0.71	0.67	0.69	0.77	0.64	0.72	0.71	0.75	0.71	0.67	0.13
**AdaBoost**	**Accuracy**	0.41	0.55	0.46	0.56	0.52	0.52	0.39	0.50	0.48	0.52	0.51	0.42	0.43	0.51	0.43	0.38	0.49	0.45	0.49	0.53	0.45	0.38	0.34	0.46	0.47	0.06
	**Precision**	0.40	0.41	0.46	0.33	0.46	0.43	0.39	0.44	0.39	0.39	0.44	0.46	0.38	0.44	0.46	0.38	0.44	0.44	0.53	0.41	0.41	0.41	0.47	0.45	0.43	0.04
	**Recall**	0.41	0.55	0.46	0.56	0.52	0.52	0.39	0.50	0.48	0.52	0.51	0.42	0.43	0.51	0.43	0.38	0.49	0.45	0.49	0.53	0.45	0.38	0.34	0.46	0.47	0.06
	**F1 Score**	0.40	0.44	0.46	0.42	0.46	0.45	0.38	0.46	0.43	0.43	0.45	0.43	0.40	0.46	0.43	0.37	0.45	0.44	0.50	0.45	0.42	0.39	0.38	0.43	0.43	0.03
**QDA**	**Accuracy**	0.28	0.22	0.31	0.28	0.24	0.42	0.23	0.41	0.20	0.21	0.24	0.28	0.32	0.25	0.58	0.32	0.31	0.30	0.36	0.26	0.34	0.24	0.56	0.28	0.31	0.10
	**Precision**	0.59	0.49	0.66	0.49	0.56	0.48	0.53	0.50	0.55	0.52	0.45	0.59	0.61	0.51	0.59	0.49	0.54	0.56	0.64	0.54	0.49	0.54	0.69	0.47	0.54	0.06
	**Recall**	0.28	0.22	0.31	0.28	0.24	0.42	0.23	0.41	0.20	0.21	0.24	0.28	0.32	0.25	0.58	0.32	0.31	0.30	0.36	0.26	0.34	0.24	0.56	0.28	0.31	0.10
	**F1 Score**	0.29	0.25	0.33	0.32	0.24	0.43	0.27	0.42	0.16	0.22	0.26	0.30	0.33	0.28	0.57	0.35	0.33	0.30	0.42	0.30	0.38	0.23	0.58	0.31	0.33	0.10
**ANN**	**Accuracy**	0.61	0.58	0.60	0.57	0.58	0.58	0.58	0.60	0.60	0.58	0.58	0.63	0.57	0.58	0.64	0.59	0.60	0.61	0.67	0.61	0.59	0.59	0.62	0.59	0.60	0.02
	**Precision**	0.69	0.42	0.56	0.54	0.48	0.54	0.34	0.69	0.67	0.62	0.61	0.54	0.60	0.52	0.60	0.52	0.52	0.60	0.64	0.57	0.56	0.62	0.58	0.58	0.57	0.08
	**Recall**	0.61	0.58	0.60	0.57	0.58	0.58	0.58	0.60	0.60	0.58	0.58	0.63	0.57	0.58	0.64	0.59	0.60	0.61	0.67	0.61	0.59	0.59	0.62	0.59	0.60	0.02
	**F1 Score**	0.52	0.43	0.48	0.45	0.44	0.44	0.43	0.48	0.49	0.44	0.45	0.53	0.46	0.44	0.54	0.46	0.46	0.50	0.59	0.48	0.48	0.48	0.55	0.48	0.48	0.04
**kNN**	**Accuracy**	0.80	0.65	0.78	0.71	0.69	0.77	0.74	0.74	0.74	0.73	0.72	0.74	0.72	0.78	0.78	0.70	0.73	0.76	0.82	0.68	0.77	0.76	0.79	0.77	0.75	0.04
	**Precision**	0.80	0.63	0.78	0.69	0.68	0.76	0.74	0.74	0.73	0.72	0.72	0.73	0.71	0.78	0.78	0.70	0.72	0.77	0.81	0.66	0.76	0.76	0.79	0.77	0.74	0.05
	**Recall**	0.80	0.65	0.78	0.71	0.69	0.77	0.74	0.74	0.74	0.73	0.72	0.74	0.72	0.78	0.78	0.70	0.73	0.76	0.82	0.68	0.77	0.76	0.79	0.77	0.75	0.04
	**F1 Score**	0.79	0.62	0.78	0.69	0.68	0.76	0.73	0.73	0.73	0.72	0.70	0.73	0.70	0.78	0.77	0.69	0.71	0.75	0.82	0.66	0.76	0.76	0.79	0.77	0.73	0.05
**XGBoost**	**Accuracy**	0.86	0.64	0.86	0.71	0.74	0.78	0.74	0.77	0.78	0.79	0.71	0.76	0.73	0.82	0.80	0.75	0.75	0.77	0.86	0.68	0.81	0.78	0.84	0.79	0.77	0.06
	**Precision**	0.87	0.62	0.86	0.72	0.74	0.79	0.74	0.78	0.79	0.79	0.72	0.76	0.73	0.83	0.80	0.76	0.75	0.77	0.85	0.66	0.82	0.79	0.84	0.79	0.77	0.06
	**Recall**	0.86	0.64	0.86	0.71	0.74	0.78	0.74	0.77	0.78	0.79	0.71	0.76	0.73	0.82	0.80	0.75	0.75	0.77	0.86	0.68	0.81	0.78	0.84	0.79	0.77	0.06
	**F1 Score**	0.86	0.58	0.85	0.69	0.72	0.77	0.72	0.76	0.76	0.77	0.69	0.75	0.72	0.81	0.78	0.73	0.73	0.75	0.85	0.64	0.80	0.77	0.83	0.78	0.75	0.07

## Data Availability

Not applicable.

## Abbreviations

The following abbreviations are used in this manuscript:

fNIRS	Functional Near-Infrared Spectroscopy
SVM	Support Vector Machine
RF	Random forest
DT	Decision Tree
QDA	Quadratic Discriminant Analysis
ANN	Artificial Neural Networks (ANN)
KNN	K-Nearest Neighbors (kNN)
